# EXPLORING VIRTUAL REALITY, VOICE INTERFACES, AND PERSONALITY TRAITS IN TECHNOLOGY INTERACTION AMONG OLDER ADULTS

**DOI:** 10.1093/geroni/igae098.1902

**Published:** 2024-12-31

**Authors:** Jimmy Reyes, Jimmy Reyes

**Affiliations:** Chair; Discussant

## Abstract

The proposed symposium highlights recent advances in addressing health disparities and technology adoption among older adults. Three noteworthy studies were undertaken by researchers spanning diverse career stages, thereby encompassing a breadth of expertise and viewpoints. The first study introduces MOTIVE, an immersive virtual exercise intervention targeting older adults who are low-income and living in urban settings. Results suggest acceptability of the intervention and support the scaling of the MOTIVE Study. Another study explores the usability of voice-assisted smart speakers among low-income minority older adults. While participants generally appreciate the hands-free interaction, challenges such as navigation difficulties and unintentional activations were noted. Customizable voice user interfaces are suggested to accommodate health conditions and speech abilities, essential for broader adoption. Contrary to stereotypes, smartphone and AI technology adoption among older adults is increasing. However, perceptions about AI may influence engagement. A pilot study investigating personality traits’ impact on AI interaction reveals extraversion predicts engagement, while agreeableness and conscientiousness affect perceived usability. Integrating personality traits into technology research protocols is crucial for understanding and enhancing older adults’ engagement. An integrated discussion, guided by the chair, will provide a comprehensive overview of recent advances in technology adoption and usability within the context of gerontology. Collectively, these scholarly discussions will offer invaluable insights into innovative interventions and technological paradigms aimed at mitigating health disparities and enhancing the quality of life among older adults.

